# Factors associated with choice of first biologic among children with juvenile idiopathic arthritis: a combined analysis from two UK paediatric biologic registers

**DOI:** 10.1186/1546-0096-12-S1-P190

**Published:** 2014-09-17

**Authors:** Rebecca Davies, Lianne Kearsley-Fleet, Eileen Baildam, Michael W Beresford, Helen Foster, Taunton R Southwood, Wendey Thomson, Kimme L Hyrich

**Affiliations:** 1 Arthritis Research UK Centre for Epidemiology, Manchester Academic Health Science Centre, The University of Manchester, Manchester, UK; 2 Hey Children’s Foundation NHS Trust, Liverpool, UK; 3 Institue of Translational Medicine (Child Health), University of Liverpool, Liverpool, UK; 4 Musculoskeletal Research Group, Institute Cellular Medicine, Newcastle University, Newcastle upon Tyne, UK; 5 Department of Paediatric Rheumatology, Institute of Child Health, Birmingham Children’s Hospital – NHS Trust and University of Birmingham, Birmingham, UK

## Introduction

The management of juvenile idiopathic arthritis (JIA) has been revolutionised through biologics such as etanercept (ETN), approved in the UK in 2002. Since that time, the use of other biologics in children and young people (CYP) has expanded. ETN is most often the first choice biologic in the treatment of JIA; however there may be occasions where ETN is not the preferred choice, for reasons of efficacy or safety.

## Objectives

The aim of this analysis was to describe the choice of first-line biologics in UK CYP with JIA and explore possible reasons behind this choice.

## Methods

Both the British Society for Paediatric and Adolescent Rheumatology Etanercept Cohort Study (BSPAR-ETN), and the Biologics for Children with Rheumatic Diseases (BCRD) study, are ongoing prospective observational cohorts, collecting detailed information on CYP starting biologics for JIA. At start of therapy, demographic and disease information is collected. Patients registered from 01/01/2010 starting a first biologic were compared between therapies using descriptive statistics. CYP starting ETN <2010 were also included to analyse changes in ETN prescribing since initial approval.

## Results

To 07/04/2014, 870 patients were recruited starting a first-line biologic (123 BCRD; 747 BSPAR-ETN (582<2010, 165≥2010) (Table 1). From 2010, CYP with systemic JIA (sJIA) were almost exclusively prescribed anakinra or tocilizumab. Choice of anti-TNF therapy was largely driven by prevalence of uveitis. Compared to ETN patients pre-2010, CYP starting ETN from 2010 had shorter disease duration, less uveitis, less sJIA, and less corticosteroid use.

**Table 1:** 

Biologic start post 01/01/2010, unless specified. *All values median(IQR) or n(%)	Etanercept [N=165]	Adalimumab [N=45]	Infliximab [N=29]	Tocilizumab [N=32]	Anakinra [N=15]	Pre-2010 Etanercept [N=582]
**Female**	109 (67%)	30 (67%)	17 (59%)	14 (44%)	11 (73%)	384 (66%)

**Age, years**	11 (8, 14)	10 (6, 14)	8 (5, 10)	8 (4, 11)	3 (2, 13)	11 (8, 14)

**Disease duration, years**	2 (1, 5)	4 (2, 6)	3 (2, 6)	1 (1, 2)	0 (0, 1)	4 (2, 7)

**ILAR Category**						
Systemic arthritis	5 (3%)	1 (2%)	1 (3%)	28 (88%)	15	70 (12%)
Oligoarthritis	39 (24%)	24 (53%)	16 (55%)	0	(100%)	117 (20%)
Polyarthritis	83 (50%)	9 (20%)	9 (31%)	3 (9%)	0	253 (43%)
Enthesitis Related Arthritis	10 (6%)	5 (11%)	2 (7%)	0	0	50 (9%)
Psoriatic arthritis	10 (6%)	5 (11%)	1 (3%)	0	0	44 (8%)
Other	18 (11%)	1 (2%)	0	1 (3%)	0	48 (8%)
					0	

**Concomitant MTX**	77 (47%)	31 (69%)	26 (90%)	28 (88%)	12 (80%)	322 (55%)

**Concomitant corticosteroids**	15 (9%)	7 (16%)	5 (17%)	23 (72%)	7 (47%)	146 (25%)

**Ever had uveitis**	7 (5%)	31 (70%)	21 (72%)	0	0	54 (11%)

**CHAQ [0-3]**	1 (0, 2)	1 (0, 1)	0 (0, 1)	1 (0, 2)	2 (1, 2)	1 (0, 2)

**JADAS-71**	13 (8, 21)	10 (7, 17)	6 (3, 12)	19 (1, 22)	23 (7, 30)	16 (9, 23)

## Conclusion

Although ETN remains the most common biologic prescribed for JIA, there has been a shift towards the use of alternative biologics, some unlicensed, largely driven by disease subtype and the presence of uveitis. This channelling of certain children towards specific therapies is important in terms of future comparative effectiveness studies and also as a guide to ongoing research priorities within rheumatology.

## Disclosure of interest

None declared.

